# Awareness of Noise-Induced Hearing Loss Related to Exposure to High-Noise Environments—Case Study: Young Adults 18 to 30 in Greece

**DOI:** 10.3390/audiolres15060171

**Published:** 2025-12-05

**Authors:** Nikolaos Trimmis, Melina Kaparou, Theodoros Tsoukalas, Panagiotis Plotas, Voula Chris Georgopoulos

**Affiliations:** 1 Department of Speech & Language Therapy, University of Patras, 26504 Rion, Greecepplotas@upatras.gr (P.P.); 2 Primary Healthcare Laboratory, School of Health Rehabilitation Sciences, University of Patras, 26504 Rion, Greece

**Keywords:** noise-induced hearing loss, awareness, young adults, prevention, auditory health

## Abstract

**Background:** Noise-induced hearing loss (NIHL) is one of the most common types of hearing impairment, even though it is preventable. However, awareness and protective behaviors among young adults remain limited. This study explored the knowledge, attitudes, and behaviors of young adults in Greece regarding exposure to high-noise environments and the risk of NIHL. **Methods:** A cross-sectional survey was conducted with 104 participants aged 18–30 years in Patras, Greece. A 27-item questionnaire was used to collect data on demographics, patterns of noise exposure, use of personal listening devices, auditory symptoms, and preventive behaviors. Descriptive statistics and chi-square tests were used to examine relationships between demographic variables and participants’ responses. **Results:** Most participants (93.3%) recognized that prolonged exposure to high noise levels can harm hearing. However, only 6.7% reported having regular hearing checks, and almost half (45.2%) had never been tested. Remarkably, 19.2% of participants experienced tinnitus, while more than half (54.8%) reported fatigue after exposure to loud sounds. Younger participants (aged 18–22 years) were significantly more likely to listen at high volumes compared to older groups (*p* < 0.05). Males reported higher rates of tinnitus and ear discomfort, whereas females more often experienced headaches. Although general awareness of NIHL was high, preventive behaviors such as using hearing protection were rarely practiced. **Conclusions:** These findings highlight the need for targeted educational campaigns and preventive screening programs to promote safe listening practices and reduce the overall prevalence of NIHL.

## 1. Introduction

Noise-induced hearing loss (NIHL) is a widespread and preventable form of sensorineural hearing loss that arises from exposure to sound levels above 85 dB SPL for extended periods [1]. The condition primarily results from irreversible damage to the cochlear hair cells, particularly those responsible for high-frequency perception, which are highly sensitive to acoustic trauma [2,3]. Studies have shown that both temporary threshold shifts (TTS) and permanent threshold shifts (PTS) can occur following prolonged or repeated exposure to high-intensity sounds, such as music at concerts, nightclubs, or through personal listening devices [4].

Globally, NIHL has been recognized as a significant public health problem. In the United States, approximately 10% of adults report permanent hearing loss attributed to noise exposure [5]. Similarly, research across Europe and Asia highlights high rates of auditory symptoms, such as tinnitus, among adolescents and young adults following recreational sound exposure [6,7,8]. For instance, Johnson et al. [6] reported that more than 70% of UK university students experienced temporary tinnitus after visiting nightclubs, while Degeest et al. [7] found that 64.8% of Belgian adolescents reported transient tinnitus after exposure to loud music, with 2.5% developing chronic symptoms. Despite this, many young people continue to underestimate the risks, with more than 70% reporting no change in their recreational listening habits even after experiencing symptoms [6].

The widespread use of personal listening devices (PLDs) has further intensified concerns. Adolescents and young adults often listen to music at high intensities, frequently exceeding safe listening levels. Byeon [8] reported that Korean adolescents who used headphones in noisy environments were 4.5 times more likely to experience hearing loss, while listening at high intensity for more than 80 min daily increased the risk by 4.7 times. Similar findings from Saudi Arabia indicated that listening to music at 90–100% of device output tripled the risk of hearing problems, although only 35% of participants were aware of these dangers [9].

Awareness of NIHL varies widely, but a consistent pattern emerges: knowledge does not always translate into preventive behavior. In Jordan, while 81% of young adults were aware that exposure to loud sounds could cause permanent hearing loss, only 9.8% reported regular use of hearing protection [10]. In the United States, Crandell et al. [11] noted that 72% of adolescents had never used hearing protection despite being aware of the risks. This disconnect between knowledge and practice underscores the need for targeted interventions.

In Greece, the issue of NIHL among young adults has received limited research attention, despite the cultural prominence of nightlife, music venues, and urban noise exposure. The city of Patras, with its large student population and vibrant recreational activities, represents a relevant context in which to examine awareness and behaviors related to NIHL. Previous studies emphasize the importance of preventive education, screening, and awareness campaigns, particularly for young adults who often fail to adopt protective measures until symptoms become persistent [6,7,8,9,10].

The present study aims to investigate awareness of NIHL among Greek young adults aged 18–30 years, focusing on their listening habits, frequency of exposure to noisy environments, prevalence of auditory symptoms, and use of preventive strategies. By examining the relationship between demographic factors, self-reported behaviors, and awareness, this study seeks to provide insights that can inform public health policies and preventive programs in Greece and beyond.

## 2. Materials and Methods

### 2.1. Study Design

This project adopted a cross-sectional descriptive design, conducted between May and September 2024 in Patras, Greece. The study employed a quantitative methodology, using a structured, self-administered questionnaire to explore young adults’ awareness, attitudes, and behaviors relating to noise-induced hearing loss (NIHL).

Patras was deliberately chosen as the study site because of its vibrant cultural and student activity, which includes large-scale social and musical events such as the Patras Carnival—one of the most well-known festivals in Greece. These lively cultural environments are often associated with high sound levels and prolonged noise exposure, making the city a particularly suitable context for investigating listening behaviors and hearing health awareness among young adults.

The focus on individuals aged 18–30 years aligns with previous literature highlighting this age group as being at greater risk for recreational NIHL, due to their frequent attendance at noisy venues and regular use of personal listening devices [6,7,8,9].

### 2.2. Participants and Recruitment

A total of 104 participants took part in the study, including 46 males (44.2%) and 58 females (55.8%), as shown in Table 1. Recruitment was based on convenience sampling from the broader community of Patras, encompassing university students, employed individuals, and young adults in leisure environments. This sampling approach allowed for the inclusion of diverse experiences related to noise exposure across educational and occupational backgrounds.

Eligibility criteria were as follows:

- Aged between 18 and 30 years;
- Resident within the Patras metropolitan area;
- No diagnosed permanent hearing impairment or history of ear surgery.

Participation was voluntary, and all respondents provided written informed consent before taking part. Ethical principles were upheld in line with the Declaration of Helsinki, ensuring the confidentiality, anonymity, and well-being of all participants throughout the research process.

### 2.3. Questionnaire Development

The survey consisted of 27 items, incorporating both closed- and open-ended questions. It was developed based on previous studies examining NIHL awareness and prevention among young adults [6,7,8,9,10]. The questionnaire was structured around five thematic domains:

1. Demographics: age, sex, education level, and employment status.
2. Exposure to Noise: frequency of attendance at noisy venues (e.g., nightclubs, concerts), use of personal listening devices (PLDs), and preferred listening volume.
3. Self-Reported Auditory Symptoms: such as tinnitus, ear pain, sound sensitivity, hearing difficulties, fatigue, or headaches following noise exposure.
4. Preventive Behaviors: including the use of hearing protection (e.g., earplugs, earmuffs), frequency of hearing checks (audiograms), and how noise levels influence leisure choices.
5. Perceptions and Attitudes: covering knowledge about NIHL, beliefs regarding its permanence, willingness to modify listening behaviors, and interest in hearing health awareness campaigns.

The questionnaire was distributed digitally through Google Forms, offering participants an accessible and time-efficient method of completion (approximately 10 min). An English translation of the full questionnaire is included in Appendix A.

Six participants completed a pilot version of the questionnaire to assess clarity and timing. Their data were included in the main analysis since only small wording edits were made to the final version.

### 2.4. Data Collection

The survey link including consent form was disseminated using a combination of mailing lists and peer-to-peer sharing networks. Participants were provided with information about the objectives of the study, as well as assurances regarding the anonymity and confidentiality of their responses.

To enhance representativeness, recruitment efforts were directed toward both students and non-students aiming to include individuals with varied everyday listening experiences.

### 2.5. Data Analysis

Data were analyzed using SPSS statistics version 26.0 (IBM Corporation, Armonk, NY, USA). Descriptive statistics (frequencies, percentages, means) summarized participant demographics, listening habits, symptoms, and awareness. Chi-square (χ^2^) tests were applied to examine associations between demographic factors (age, sex, education, occupation) and outcome variables, such as:

- Exposure to high-noise environments;
- Presence of auditory symptoms;
- Use of preventive behaviors;
- Attitudes toward NIHL awareness.

Significance was set at *p* < 0.05. Where appropriate, data were stratified by age group (18–22; 23–26; 27–30) and sex.

## 3. Results

The educational background of the respondents showed a diverse distribution across all levels of education. Among the 104 young adults surveyed, the largest proportion were university graduates (30.8%), followed by high school graduates (27.9%). A further 20.2% reported holding a Master’s degree, while 11.5% had completed post-secondary vocational training (in Greek “IEK” or equivalent). Smaller groups included secondary school students (5.8%), junior high school graduates (2.9%), and only 1.0% of respondents had obtained a PhD. This distribution shows a diverse educational profile, though it is somewhat weighted toward participants with higher education. The predominance of university and postgraduate graduates likely reflects the strong student population in Patras, the main setting of this study.

### 3.1. Noise Exposure

Participants were first asked to describe the general noise level of their living environment. Most characterized their surroundings as Quiet, while over one third reported Noisy or Very noisy conditions (Table 2).

Most respondents reported regular use of sound-producing devices. Nearly two thirds (60.6%) indicated that they used devices such as televisions, mobile phones, or headphones “often” or “always.” With respect to volume settings, 76.9% of participants reported listening at high levels. Volume level categories (“Very low,” “Low,” “Medium,” “High,” “Very high”) reflect participants’ self-perceived loudness settings on their personal listening devices and represent subjective estimates rather than measured decibel values. High-volume listening was particularly common among the youngest age group (18–22 years). These findings highlight that a considerable proportion of young adults are exposed to potentially harmful listening practices (Table 3).

### 3.2. Noise Reported Symptoms

**Figure 1:**
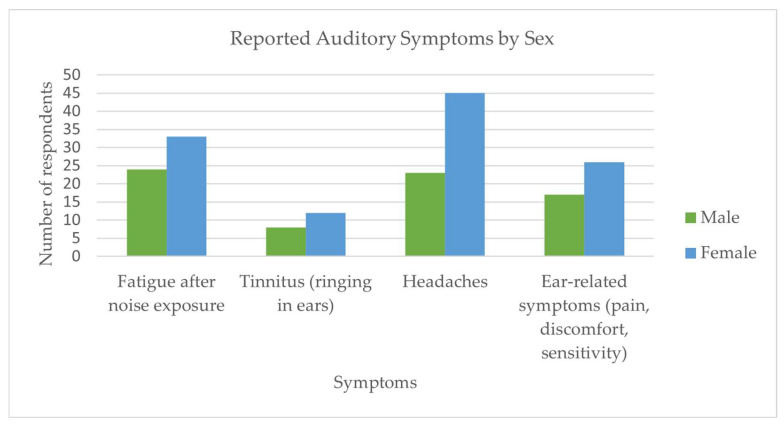
Reported auditory and related symptoms by sex among young adults (*n* = 104). Participants could select more than one symptom; therefore, totals exceed the number of respondents per sex.

Noise-related symptoms were frequently reported. More than half of the participants (54.8%) experienced fatigue after exposure to loud sounds, and nearly one in three (29.8%) reported frequent headaches. Tinnitus was present in 19.2% of respondents, while 21.2% experienced other ear symptoms such as pain or a sensation of pressure or blockage in the ears. Notably, 17.3% had been advised by others to undergo a hearing check, and 23.1% perceived a reduced tolerance to loud sounds over time. Both males and females most frequently reported experiencing fatigue after exposure to loud noise, as well as headaches. Overall, females reported higher rates for all symptom categories (Figure 1). Slightly more than one-third of participants reported no symptoms at all.

Further analysis showed that 31.7% of respondents indicated they had never considered fatigue after loud sound exposure, and only 13.5% stated they had not experienced such fatigue. Headaches were also common: 35.6% reported experiencing them once or twice per month, and 34.6% reported them rarely or never. However, 16.3% experienced headaches at least once per week, and 4.8% more than twice per week. When asked about ear-related symptoms, the majority (58.7%) reported no symptoms. Among the remainder, sound hypersensitivity (17.3%) and tinnitus (10.6%) were the most common, while smaller proportions reported hearing loss (5.8%), ear pain (4.8%), or sound distortion (2.9%). These findings indicate that while a significant share of young adults report some form of auditory or related discomfort, nearly 40% already perceive at least one symptom associated with potential auditory strain or damage.

### 3.3. Preventive Behaviors

Engagement in preventive hearing health behaviors was limited. Although 40.4% of participants had undergone an audiogram at least once, only 6.7% reported regular hearing checks. Nearly half (44.2%) agreed with the statement “I can tolerate loud sounds, so I do not worry about hearing protection,” reflecting a barrier to preventive action. Just over one third (34.6%) reported selecting leisure venues based on noise levels, while the majority did not consider noise exposure when making such choices.

### 3.4. Awareness and Attitudes

**Figure 2:**
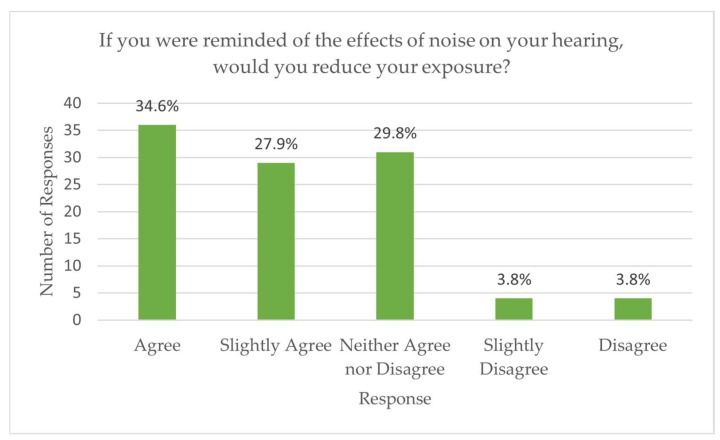
Responses to the question “If you were reminded of the effects of noise on your hearing, would you reduce your exposure?”.

**Figure 3:**
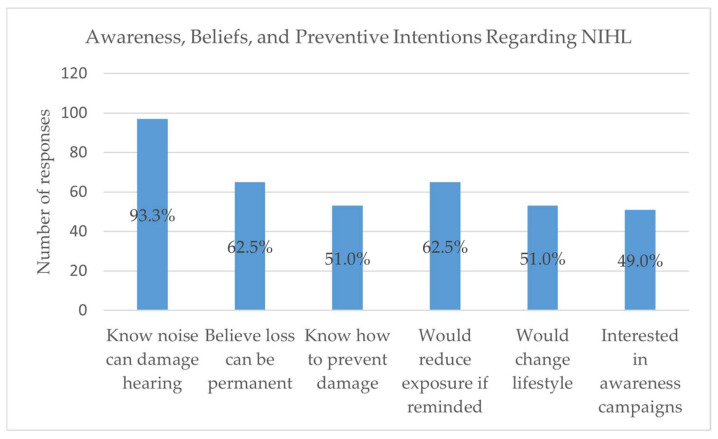
Awareness, beliefs, and preventive intentions regarding noise-induced hearing loss (NIHL).

Awareness of the risks associated with noise exposure was high. Most respondents (93.3%) recognized that prolonged exposure to high noise levels can damage hearing, and 62.5% understood that such hearing loss may be permanent. However, only half (51.0%) reported knowing how to prevent hearing damage. Encouragingly, the majority expressed positive attitudes toward prevention: 62.5% indicated they would reduce noise exposure if reminded of the risks (Figure 2), 74.0% reported a willingness to make lifestyle changes, and 86.5% expressed interest in awareness and prevention campaigns (Figure 3).

### 3.5. Education Level and Awareness/Behaviors

When stratified by education level, descriptive patterns suggested that lower-educated participants (high school and vocational graduates) reported tinnitus more frequently, whereas higher-educated groups (university and postgraduate graduates) were more likely to have undergone a hearing test. Nonetheless, awareness of noise-induced hearing risks was consistently high across all education groups.

Chi-square analyses did not reveal any statistically significant associations between education level and awareness (χ^2^, *p* = 0.102), preventive knowledge (*p* = 0.916), tinnitus (*p* = 0.457), or audiogram use (*p* = 0.232). This indicates that, within this sample, educational attainment was not a strong predictor of awareness, symptoms, or preventive actions.

## 4. Discussion

This study investigated the awareness of noise-induced hearing loss (NIHL), exposure to high-noise environments, and preventive behaviors among young adults in Greece. The results demonstrate that while awareness of the risks associated with noise exposure is relatively high, preventive behaviors remain limited. A considerable proportion of participants reported auditory symptoms such as fatigue, tinnitus, and headaches, with females more likely to report headaches and males more likely to report tinnitus and ear discomfort. Despite this, fewer than half of the participants had ever undergone an audiogram, and the use of hearing protection was rare.

The prevalence of symptoms such as tinnitus (19.2%) and reduced tolerance to loud sounds (23.1%) aligns with findings from international studies among young adults, where tinnitus prevalence is reported between 15 and 31% [1,2,12]. Fatigue and headaches after noise exposure, reported by more than half of participants, are consistent with evidence that environmental noise has both auditory and non-auditory health consequences, including stress and sleep disturbances [3,4].

Although nearly all respondents (93.3%) acknowledged that prolonged noise exposure can damage hearing, only half (51.0%) knew specific preventive strategies. This discrepancy between awareness and behavior has been widely documented in the literature [5,6]. For example, studies in Northern Europe and the United States similarly report that while knowledge of NIHL is widespread, the use of earplugs in noisy recreational settings remains below 20% [7,8]. In a UK study, although 75% of young adults recognized that loud music may cause permanent tinnitus, exposure to a tinnitus-health message did not significantly increase their intentions to adopt protective behaviors, indicating that awareness alone may not suffice to change listening habits [13]. Cultural and behavioral factors—such as the perception that “tolerance” to noise reduces risk, which was endorsed by 44.2% of our sample—may contribute to this gap. Such beliefs have also been observed in other Mediterranean populations, suggesting that regional attitudes toward leisure noise may influence preventive practices [9].

Even though this study did not find a significant relationship between education level and listening behaviors, other research has shown that individuals with lower educational attainment are more likely to engage in risky listening habits, such as listening to music at high volumes via headphones, suggesting that socioeconomic factors may subtly influence preventive hearing practices [14].

Similar patterns were reported by Fragoulia et al. [15], who found that although Greek adolescents demonstrated relatively high awareness of hearing loss, their engagement in preventive practices remained low. These findings align with the present study, suggesting that educational interventions alone may not be sufficient to alter behavior. Recent evidence from Söylemez et al. [16] further highlights the psychosocial dimensions of hearing-related behaviors: adolescents with hearing loss who used hearing aids or cochlear implants showed elevated levels of smartphone dependence and fear of missing out (FoMO). Together, these studies emphasize that hearing health attitudes and behaviors are influenced by both cognitive and social factors, reinforcing the need for multidimensional awareness programs.

### 4.1. Implications for Public Health and Audiology Practice

These findings highlight the need for targeted public health interventions in Greece. Current evidence emphasizes the need for stricter adherence to safe exposure thresholds, as recent research indicates that chronic exposure above 70 dB SPL may still contribute to hearing loss, challenging the long-standing 85 dB for 8 h occupational guideline [17]. Awareness campaigns were strongly supported by participants (86.5%), indicating a readiness to engage with educational efforts. Given that young adults frequently use personal listening devices at high volumes, strategies such as smartphone-based warnings, social media campaigns, and integration of hearing conservation messages into university health services may be effective. Audiologists and other hearing health professionals should also promote regular hearing screenings for this age group, as early identification of hearing changes can improve long-term outcomes.

### 4.2. Limitations

A strength of this study is the use of a structured questionnaire specifically designed to capture exposure, symptoms, preventive behaviors, and awareness in young adults. The sample size (*n* = 104) provided sufficient variability across age and sex groups to identify trends. However, the study has limitations. First, it relied on self-reported data, which may be subject to recall or reporting bias. Second, participants were primarily from urban areas, potentially limiting generalizability to rural populations. Finally, the cross-sectional design precludes conclusions about causality between noise exposure and reported symptoms.

## 5. Conclusions

This study highlights that although young adults in Greece are largely aware of the risks of noise-induced hearing loss, preventive behaviors remain insufficient. A substantial proportion of participants reported symptoms such as tinnitus, fatigue, and headaches, yet few had undergone regular hearing checks or used protective measures. The persistence of beliefs such as “tolerance” to loud sounds suggests that awareness does not always translate into protective action.

These findings underline the importance of targeted public health campaigns and early preventive strategies. Audiologists and hearing health professionals should be actively involved in raising awareness, encouraging regular screening, and promoting the consistent use of hearing protection in high-noise environments. Educational interventions tailored to young adults, particularly those using personal listening devices at high volumes, may help close the gap between knowledge and behavior.

Future efforts should extend beyond awareness to focus on behavioral change and sustainable prevention, ensuring that young adults adopt habits that safeguard their hearing across the life course.

## Figures and Tables

**Table 1:** Participant Demographics.

Characteristic	Category	*n*	%
Sex	Male	46	44.2
	Female	58	55.8
Age group (years)	18–22	23	22.1
	23–26	10	9.6
	27–30	71	68.3

**Table 2:** Perceived environmental noise level among participants (*n* = 104).

Noise Level	Number of Responses	% of Responses
Very quiet	2	1.9
Quiet	53	51.0
Not quiet	11	10.6
Noisy	33	31.7
Very noisy	5	4.8

**Table 3:** Reported listening volume levels by age group (*n* = 104).

Age Group (Years)	High Volume (*n*, %)	Low Volume (*n*, %)	Very Low Volume (*n*, %)
18–22	20 (87.0%)	3 (13.0%)	0 (0.0%)
23–26	7 (70.0%)	2 (20.0%)	1 (10.0%)
27–30	53 (74.6%)	16 (22.5%)	2 (2.8%)

Note. No participants selected the “very high” or “medium” volume options.

## Data Availability

The data presented in this study are available on request from the corresponding author due to privacy or ethical restrictions.

## Abbreviations

The following abbreviations are used in this manuscript:

NIHL	Noise-Induced Hearing Loss
PLD	Personal Listening Device
WHO	World Health Organization

## Appendix A

The 27-item Questionnaire used in thi study, translated in English language is presented below.

### Awareness of Noise-Induced Hearing Loss Among Young Adults

The objective of this questionnaire is to investigate the awareness of young people aged 18–30 regarding exposure to environmental noise. The questionnaire is anonymous and takes approximately 10 min to complete. All questions with an asterisk are required.

1. What is your sex? (Male/Female/Prefer not to say)
2. What is your age? * (18–22/23–26/27–30)
3. What is your highest level of education? * (Secondary school Student/Junior High School Graduate/High School Graduate/Vocational School (IEK) or Equivalent Graduate/University (Undergraduate) Graduate/Master’s Degree/PhD (Doctoral Degree))
4. How would you characterize the environment you live in? * (Very quiet/Quiet/Not quiet/Noisy/Very noisy))
5. How often do you use sound-producing devices (TV, mobile phone, headphones, speakers, etc.)? * (Never/Rarely/Sometimes/Often/Always)
6. When you use these devices, how many hours per day do you usually listen? * (<1 h/1–3 h/3–5 h/>5 h)
7. At what volume level do you usually set your devices? * (Very low/Low/Medium/High/Very high)
8. How often do you attend nightclubs, concerts, or other high-noise recreational venues? * (Never/Rarely/Sometimes/Often/Very often)
9. Do you use personal listening devices (e.g., headphones, earbuds) in noisy outdoor environments? * (Never/Rarely/Sometimes/Often/Always)
10. Do you currently work or study in the health sector? * (Yes/No)
11. If yes, do you believe that your profession increases your awareness about hearing health? (Yes/No/Not sure)
12. Do you experience ringing in the ears (tinnitus) after exposure to loud noise? * (Never/Rarely/Sometimes/Often/Always)
13. Do you experience pain in the ears after exposure to loud noise? * (Never/Rarely/Sometimes/Often/Always)
14. Do you experience hearing difficulties after being in noisy environments? * (Never/Rarely/Sometimes/Often/Always)
15. Do you experience increased sensitivity to sounds (hyperacusis) after noise exposure? * (Never/Rarely/Sometimes/Often/Always)
16. Do you experience a feeling of fatigue after being in noisy environments? * (Never/Rarely/Sometimes/Often/Always)
17. Do you experience headaches after being in noisy environments? * (Never/Rarely/Sometimes/Often/Always)
18. Have you ever been medically diagnosed with a hearing problem? * (Yes/No)
19. Do you use hearing protection (e.g., earplugs, earmuffs) in noisy environments? * (Never/Rarely/Sometimes/Often/Always)
20. How often do you check your hearing (e.g., audiogram)? * (Never/Rarely/Occasionally/Regularly)
21. Would you avoid a recreational activity (club, concert, bar) because of the high noise level? * (Yes/No/Sometimes)
22. Have you ever lowered the volume of your device because you felt discomfort in your ears? * (Yes/No/Sometimes)
23. Do you know that exposure to high levels of noise over time can cause permanent hearing damage? * (Yes/No/Not sure)
24. Do you believe that hearing loss from noise exposure is reversible? * (Yes/No/Not sure)
25. Do you think young adults are adequately informed about the risks of noise-induced hearing loss? * (Yes/No/Not sure)
26. Would you reduce your exposure to loud noise (e.g., lower volume, attend fewer noisy venues) if reminded of the risks to your hearing? * (Yes/No/Maybe)
27. Please write any comments you would like to share about noise exposure and hearing health. (Free text response)
